# Verbal and visual stimulation effects on rectus femoris and biceps femoris muscles during isometric and concentric

**DOI:** 10.1186/1755-7682-6-38

**Published:** 2013-10-08

**Authors:** Sidney B Silva, Luiz Carlos de Abreu, Vitor E Valenti, Daniel V Nogueira, Éder R Moraes, Vilma Natividade, Paulo Rogério Gallo, Dafne Herrero, Patrícia M D Zacaro

**Affiliations:** 1 Departamento de Morfologia e Fisiologia, Faculdade de Medicina do ABC. Av. Príncipe de Gales, 821. Príncipe de Gales, 09060-650 Santo André, SP, Brasil; 2 Departamento de Fonoaudiologia, Faculdade de Filosofia e Ciências, Universidade Estadual Paulista, UNESP. Av. Higyno Muzzi Filho, 737. Câmpus Universitário, 17525-900 Marília, SP, Brasil; 3 Universidade do Vale do Paraíba. Av. Shishima Hifumi, 2911 Urbanova, 12244-000 São José dos Campos, SP, Brasil

**Keywords:** Muscles, Electromyography, Knee, Auditory stimulation, Visual stimulation

## Abstract

**Background:**

Coactivation may be both desirable (injury prevention) or undesirable (strength measurement). In this context, different styles of muscle strength stimulus have being investigated. In this study we evaluated the effects of verbal and visual stimulation on rectus femoris and biceps femoris muscles contraction during isometric and concentric.

**Methods:**

We investigated 13 men (age =23.1 ± 3.8 years old; body mass =75.6 ± 9.1 kg; height =1.8 ± 0.07 m). We used the isokinetic dynamometer BIODEX device and an electromyographic (EMG) system. We evaluated the maximum isometric and isokinetic knee extension and flexion at 60°/s. The following conditions were evaluated: without visual nor verbal command (control); verbal command; visual command and; verbal and visual command. In relation to the concentric contraction, the volunteers performed five reciprocal and continuous contractions at 60°/s. With respect to isometric contractions it was made three contractions of five seconds for flexion and extension in a period of one minute.

**Results:**

We found that the peak torque during isometric flexion was higher in the subjects in the VVC condition (p > 0.05). In relation to muscle coactivation, the subjects presented higher values at the control condition (p > 0.05).

**Conclusion:**

We suggest that this type of stimulus is effective for the lower limbs.

## Background

Several factors were suggested to influence strength and muscle activation, such as emotional and cognitive [1-4]. In order to achieve a maximum human potential in sports and to develop new techniques and to improve rehabilitation, the use of stimuli such as visual and verbal encouragement are used to develop at the best progression [5,6].

The purpose of these stimulations is to increase muscle performance and accuracy of certain movements in which athletes and people during the rehabilitation process are performing as well as to improve the state of muscles synergism by coactivation [6-9]. This coactivation were studied mainly in the knee joint that has a high propensity to injury, mainly due to failure of the ligaments in the activation of muscles involved in the joint stabilization [10].

The sensory stimuli are a target of extensive research in recent years in rehabilitation and sports areas in order to increase muscle strength and also to stimulate brain areas not often used during the execution of voluntary movements. This type of stimulation usually aims to increase the response to training, improve the physical capabilities as well as decreasing the rehabilitation time. During the rehabilitation process there are times when having the ability to unload a joint can assist with faster healing and return to play [6,11].

Since the introduction of the isokinetic movement concept by Hislop and Perrine [12] in 1967, many researches tried to figure out the best way to encourage subjects to obtain the maximum performance during isometric and isokinetic contractions. The combination of techniques such as electromyography may help to assess muscle function mainly in search of muscle patterns.

The knee is a very important joint involved in the rehabilitation process [12,13,15]. In several studies, visual proprioceptive stimuli, such as imagery practice and visual computer feedback training, have been shown to be effective mainly in motor response and muscle strength [14,15]. Another investigation reported that air assault soldiers presented more dangerous landing biomechanics when visual input was removed [15,16]. Furthermore, previous studies evaluated the effects of auditory stimulation on lower limb movements [16-18]. Taken together, visual and auditory stimulation mechanisms are suggested to be strongly related to lower limb movements.

Although the literature reports several studies regarding the use of verbal and visual stimuli, there is no consensus on the best way to stimulate the individual during isometric and isokinetic tests in relation to muscle strength. Another point to note is the lack of studies which assessed the effects of verbal and visual stimuli on muscle coactivation. Therefore, we aimed to evaluate the effects of verbal and visual stimulation on rectus femoris and biceps femoris muscles contraction during isometric and concentric.

## Method

### Study population

We analyzed 13 healthy male and sedentary volunteers, with no history of knee injury, lower limb musculoskeletal injury, neurological, auditory or visual deficits, aged between 18 and 32 years old (23.1 ± 3.8 years old). The study was approved by the Ethic Committee in Research of our Institution (in accordance with Resolution No. 196/96 of the National Health Council) (Number 0255/08). The volunteers performed four tests with an interval of seven days between the data collection, in each test a stimulus was provided. The sequence of stimuli was randomized for each volunteer: NVVC: No verbal or visual command (Control); VVC: With verbal and visual command; VeC: Verbal command; ViC: Visual command.

### Isokinetic dynamometer

To obtain the data on muscle performance, we used the Computerized Isokinetic Biodex, Biodex Multi-joint System model and its accessories.

### Electromyographic evaluation

For the acquisition of electromyographic (EMG) signal we used an EMG System from Brazil Ltda., which consists of eight channels and surface electrodes of the active and bipolar types for capturing the electrical activity of muscles. Analog signals were pre-amplified in the differential electrode in 20 times and then it was filtered with band pass filter of 20 to 500 Hz and amplified again in 100 times. The total final amplification was 2000 times. To perform the procedures cited above, we used the AqDados 4 software, which allows the initial treatment of the raw data and visualization of data collection time. The sampling frequency used for collection was 2000 Hz.

### Electrodes

We used the active type disk-shaped electrodes with silver in the center with a diameter of 1 cm of catchment and polyurethane in the rest (1.4 cm), in a total of 2.4 cm of diameter and 3 mm in height. The electrodes were placed on the motor point of the rectus femoris muscle and on the long head of the biceps femoris muscle.

### Procedures

Before the collection the volunteers performed a five minutes exercise on a cycle ergometer and stretching exercises in the lower limbs in order to avoid complications during the collection of data [19]. After warming-up the volunteers were positioned in the Biodex chair with an inclination of 85°. The axis of the dynamometer was aligned with the axis of rotation of the knee. The lateral epicondyle of the femur, pelvis and trunk were fixed by belts attached diagonally and the volunteers were instructed to cross their arms during the tests.

The gravity correction was performed according to the instructions of the equipment, i.e., as the largest action of gravity. Data were collected by the same investigator and the limb evaluated was the dominant limb. Data collection was divided into two phases, isometric and isokinetic, the two phases were performed on the same day according to the stimulus provided.

In the isometric phase, knee flexion was performed in an angle of 60°, because some authors reported that this angle provides the greatest torque generation [20]. The volunteers performed three sets of flexion/extension in five seconds for each contraction and 60 seconds of rest between contractions.

In the isokinetic phase the test was conducted at 60°/s, the volunteer performed five maximal contractions of flexion/extension. The range of motion was 70°, from 90° of flexion to 20° extension.

The subjects performed the exercises in the following conditions:

NVVC: this test was considered the control because the volunteers did not receive stimuli (without verbal or visual command); VVC: volunteers were verbally encouraged to achieve maximum strength and also the computer monitor was positioned in front of the volunteer and the subject was told to observe his performance during the test (verbal and visual command). VeC: during this test it was provided only verbal stimulation and the volunteer was blindfolded to avoid other stimuli (verbal command). ViC: in this test the volunteers were instructed to observe on the computer monitor its performance through the supplied graphic software (visual command).

Visual, information was based on EMG data while verbal stimulation was based on the following sentence: “Go ahead”.

### Normalization

The data of isometric and isokinetic peak torque were normalized according to the body mass of each subject. For EMG data, we used a protocol well accepted in the literature, in which the RMS value of the electromyographic activity of the antagonist muscle contractions is normalized as a percentage of agonist activity of this muscle [20-22].

### Statistical analysis

Standard statistical methods were used for the calculation of means and standard deviations. Normal Gaussian distribution of the data was verified by the Shapiro-Wilk goodness-of-fit test (z value >1.0). For parametric distributions, we applied the ANOVA for repeated measures followed by the Bonferroni post-test and for nonparametric distributions we used the Friedman test followed by the Dunn’s test. Differences were considered significant when the probability of a Type I error was less than 5% (p > 0.05). We used the statistical program *Matlab 6*.*1*®.

## Results

Table 1 displays values of the statistical analysis of peak torque during isometric and concentric flexion and extension. We observe that the subjects in the VVC condition presented significant increased values compared to the control condition regarding isometric flexion.

**Table 1:** Values of the statistical analysis of peak torque during concentric and isometric flexion and extension

**Peak torque(N)**	**NVVC**	**VVC**	**Vec**	**ViC**
Extension	201 ± 17.2	201 ± 45.6	205 ± 31.8	218 ± 30.5
Flexion	97 ± 14.5	96 ± 20.6	98 ± 20.8	100 ± 16.1
Isometric Extension	243.0 ± 28	253.4 ± 32.5	247.2 ± 30.9	246.6 ± 29.1
Isometric Flexion	105.0 ± 13.1	114.0 ± 12.3*	112.7 ± 17.6	110.9 ± 13.8
Porcentage of Isokinetic movement 60º	79.0 ± 7	80.5 ± 3.4	80.1 ± 4.2	81.2 ± 4

NVV: No verbal or visual command (Control); VVC: With verbal and visual command; VeC: Verbal command; ViC: Visual command. *p > 0.05: Vs. NVVC.

**Figure 1:**
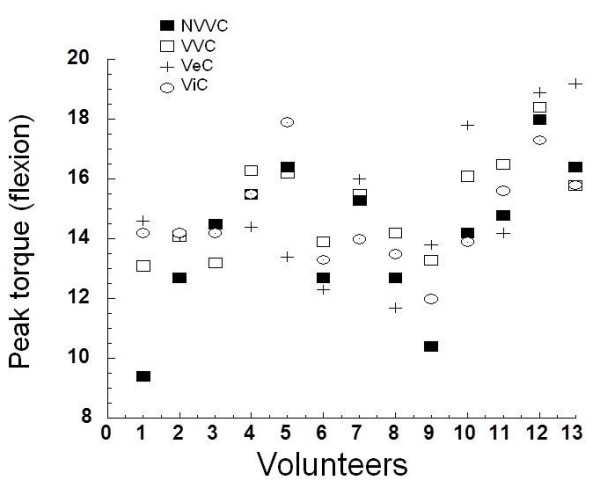
**Normalized flexor isometric peak torque at 60º**/**s.** NVV: No verbal or visual command (Control); VVC: With verbal and visual command; VeC: Verbal command; ViC: Visual command.

We observe in Figure 1 the values of the peak torque during knee flexion in each volunteer. It is noted that although the peak torque values for the control condition tended to be lower for the most of the subjects, there was no statistical differences.

**Figure 2:**
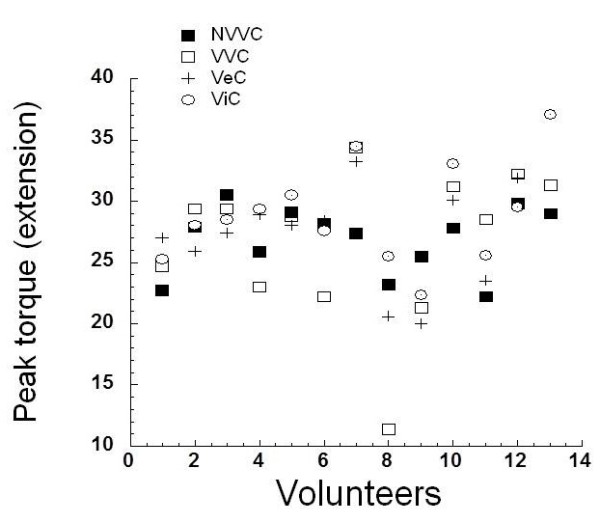
**Normalized extensor Isometric peak torque at 60º**/**s.** NVV: No verbal or visual command (Control); VVC: With verbal and visual command; VeC: Verbal command; ViC: Visual command.

We found no significant differences in the analysis of the extension peak torque. The extension peak torque values in the control situation presented similar characteristics to the flexor peak torque. This is clearly evident in Figure 2, where we note the peak torque of each volunteer.

**Figure 3:**
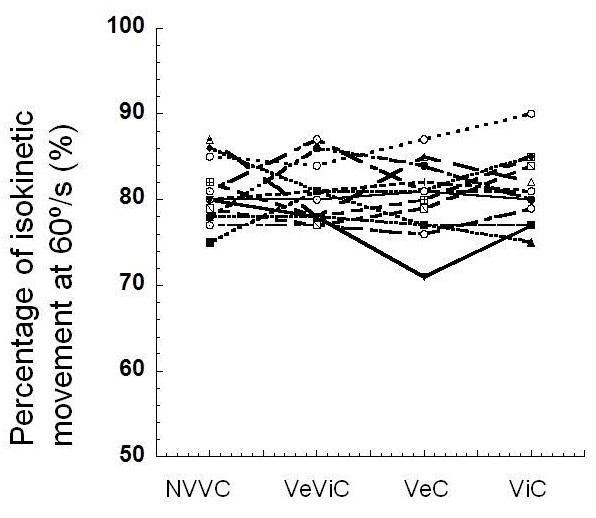
**Percentage of isokinetic movement at 60º**/**s.**

**Figure 4:**
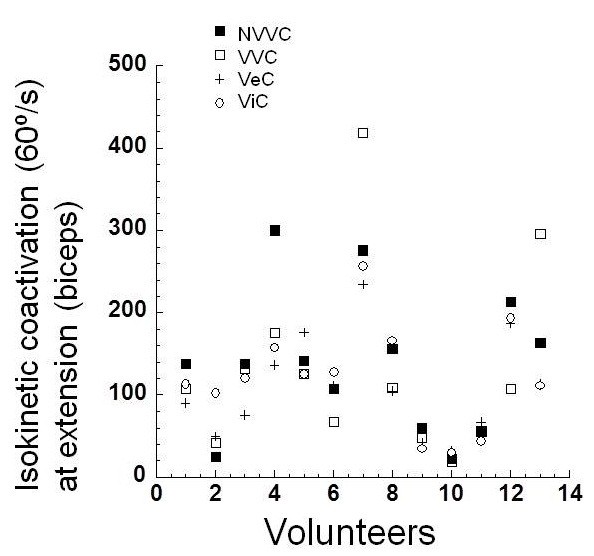
**Coactivation of the biceps muscle in extension during isokinetic contraction at 60º**/**s.**

When we analyzed the percentage of isokinetic movement at 60º/s we found no differences between the four conditions (Figures 3 and 4).

According to Table 2, there was no difference regarding muscle coactivation in flexion and extension at 60º/s. The coactivation of the biceps femoris muscle during extension at 60º/s tended to be higher in the control condition. However, it did not reach statistical significance. The rectus femoris muscle also presented the same behavior of the biceps femoris muscle at 60º/s (Table 2).

**Table 2:** **Values of the statistical analysis for muscle coactivation in flexion** (**rectus femoris**) **and extension** (**biceps**) **during isokinetic movements at 60**°/**s and isometric contraction**

**Muscle coactivation**	**NVVC (%)**	**VVC (%)**	**VeC (%)**	**ViC (%)**
Extension (Bíceps) (60º/s)	147.1 ± 94	134.6 ± 88.4	121.1 ± 55	88.7 ± 4.8
Flexion (Retus Femoris) (60º/s)	81.8 ± 17.4	75.8 ± 24.9	133.2 ± 75.4	88.4 ± 17.3
Extension (Bíceps) (180º/s)	116.7 ± 78	118 ± 70.7	117.8 ± 68.7	128.5 ± 52.3
Flexion (Retus Femoris) (180º/s)	59.1 ± 54.3	46.2 ± 34.8	46.1 ± 49.1	58.7 ± 51.8
Isometric extension (Bíceps)	108.5 ± 48.1***	108.0 ± 75.5	84.2 ± 29.5	93.5 ± 40.3
Isometric flexion (Retus Femoris)	11.1 ± 6.3	10.2 ± 2.2	12.4 ± 12.5	13.3 ± 12.3

NVVC: No verbal or visual command (Control); VVC: With verbal and visual command; VeC: Verbal command; ViC: Visual command. *p > 0.05: Vs. VeC and ViC.

In relation to muscle coactivation, we found difference between NVVC and VeC conditions and between NVVC and ViC conditions during isometric extension. The subjects presented higher values at the control condition (p > 0.05) (Table 2).

## Discussion

During the training process, as well as in rehabilitation, the goal is to increase muscle strength, advocating the use of sensory stimuli. Thus, we evaluated the effects of visual and verbal stimuli on isometric and isokinetic exercise. We investigated sedentary volunteers because they are not used to develop strength workouts or fatigue and we expected that the stimuli could be more reliable, and subjects who engage in physical activity have also better learning during the execution of the exercises and so the analysis of coactivation could also be affected by neuromuscular adaptations. Our results showed differences in relation to the data found by other researchers regarding the effect of verbal and visual stimuli during isometric and isokinetic contractions.

In our study, only the flexor peak torque during isometric contraction presented differences between the control condition and the group with verbal and visual stimuli. For the other stimuli we observed no changes in peak torque, both during flexor and extensor isometric exercise. Peacock et al. [23] found similar results, however, they evaluated only knee extension.

According to Smidt and Rogers [19], the angle of greater activity of the isometric strength is around 45º to 60º and the flexor peak torque is higher around 60º, while the best angle for extension is around 45º. We chose to evaluate both flexor and extensor peak torque in the range of 60º. This amplitude may have favored the flexor peak torque, although no change in flexor peak torque occurred in relation to other stimuli. It would be important when comparing flexion to extension, however, the stimulus position did not affect the results.

Rasch and Pierson [24] and Berger [25] investigated the effects of visual stimulus and found an increase in peak torque. They evaluated elbow and hands, respectively. Also McNair et al. [26] and Johansson et al. [27], when assessed verbal stimulation, found an increase of 5% and 8%, respectively, in peak torque when the stimulus was provided. In their work the authors evaluated the isometric peak torque of the flexor and extensor muscles of the elbow, respectively. Johansson et al. [27] also investigated the effects of verbal stimulus intensity and found that when the stimulus was performed aloud, the subjects developed a higher force during exercise, as in our study. However, no change in the peak torque was found.

Campenella et al. [5] evaluated verbal and visual stimuli effects on isokinetic peak torque at 60º/s. Their results showed an increase in peak torque in verbal and visual stimuli together and only in the visual stimulus. Nevertheless, verbal stimulation had no significant effects. They reported difficulty in standardizing verbal stimulation for volunteers, and because of this the volunteers did not develop adequate strength during the test. In our study, no differences were found in the evaluation of the stimuli. In the study of Campenella et al. [5], the control condition was used as the first test, which may have influenced the motor learning. On the other hand, Fernández-Ruiz et al. [28] reported that the motor learning starts from the first exercise that the individual performs, regardless of the information provided to the individual. To minimize this type of influence on the results we chose to randomize the sequence of collection.

A variable that could help to explain the slight change in peak torque is the percentage of isokinetic movement. If the subject did not alter the peak torque after some stimuli, the percentage of isokinetic movement could show a decline, considering that the volunteers did not exert maximum force throughout the movement. According to our findings, the percentage of isokinetic movement was not changed. Tortoza et al. [29] demonstrated that it was smaller at 180º/s, which indicated that the increase in the speed decrease the percentage of isokinetic movement.

Based on our data, the peak torque and the percentage of isokinetic movement were not changed after visual or verbal stimulation, separate or alone. At first it was hypothesized that the stimuli could have increased muscle coactivation and, therefore, the individual could not increase strength. Kellis and Baltzopoulos [30] reported that coactivation of the muscles surrounding the knee reduce the joint displacements in order to prevent injury, and it decreases muscle strength. Our findings showed no differences in relation to the different stimuli to coactivation on the isokinetic contractions at 60º/s. Therefore, we may suggest that muscle coactivation was not responsible for the lack of change in peak torque in a variety of stimuli, indicating that subjects developed maximum strength in all tests regardless of the stimulus, with the same degree of muscle coactivation.

An interesting finding to be emphasized in relation to coactivation was found in isometric contractions. We found that when verbal and visual stimuli were provided separately during knee extension, the biceps femoris muscle was less active and the peak torque did not change during the two stimuli. It indicates that although the extensor peak torque remains unchanged the biceps femoris muscle used lower number of muscle fibers to maintain the same stability in the joint. This response during muscle activation was not predominant in flexion as well as in extension. McNair et al. [26] evaluated the electrical activity only in the biceps femoris muscle and found no changes in electrical activity, while peak torque increased. Gottlieb et al. [31] reported that coactivation has central and peripheral mechanisms, hence, it is believed that the angle of movement used to assess the knee joint may have resulted in less stress on joint receptors, leading to a lower coactivation for the both stimuli during extension.

This hypothesis is relevant because the flexor peak torque increased, however, the rectus femoris coactivation did not change when the verbal and visual stimuli were delivered together. Thus, the neural control of coactivation may have been higher in the control of muscle contraction in order to stabilize the joint with the same degree of coactivation to a greater peak torque during the exercise.

Giray and Ulrich [32] reported that sensory stimuli are part of the intrinsic regulation of muscles activation. They suggested that muscle activation could be modulated according to the intensity. In our study we found conflicting results regarding the coactivation of muscle when applied verbal and visual stimuli. It was expected that this coactivation would be decreased in both flexion and extension, however, it was not observed. The decrease in coactivation occurred only in the extension during only one separate stimuli. We suggest that it may be involved with an increased charge of stimuli delivered to the brain.

We reported different results for isometric and isokinetic muscle contractions. The difference between the two types of contraction was reported by Rosa and coworkers [33]. The authors observed that in long term isokinetic exercises present increased effectiveness compared to isometric exercises for muscle strength and pain in patients with knee osteoarthritis. Another group [34] reported different neuromuscular fatigue features regarding the different types of muscle contraction. Isometric and concentric contractions displayed different neuromuscular fatigue profiles while eccentric activity was largely fatigue resistant. This mechanism may be indicated as one factor to explain our findings.

Some points in this study are worth to be raised. Considering that individuals develop the same strength, percentage of motion and isokinetic muscle coactivation as well, we suggest that the simple fact of orient them before testing could motivate them to develop maximum strength regardless of the stimulus. We suggest further studies with additional protocols based on this matter. We measured the moments in a Biotex dynamometer, however, the alignment of these two axes at rest (i.e. inactive condition) does not guarantee correct measurements during isometric or isokinetic contractions because the alignment of the two axes changes.

## Conclusion

Both verbal and visual stimuli influenced the isometric peak torque during knee flexion. The verbal and visual stimuli alone were more effective in controlling the coactivation of the biceps femoris muscle during isometric contraction extension without changing the muscle strength. Therefore, our data suggest that this type of stimulus is effective for the lower limbs.

## Competing interest

All authors declare that they have no competing interest.

## Authors’ contribution

SBS, LCA, VEV, DVN, ERM, VN, PRG, DH, PMDZ participated in the acquisition of data and revision of the manuscript. SBS, LCA, VEV, PMDZ conceived of the study, determined the design, performed the statistical analysis, interpreted the data and drafted the manuscript. All authors read and gave final approval for the version submitted for publication.
